# Multiple primary malignancies involving lung cancer

**DOI:** 10.1186/s12885-015-1733-8

**Published:** 2015-10-14

**Authors:** Feng Li, Wen-Zhao Zhong, Fei-Yu Niu, Ning Zhao, Jin-Ji Yang, Hong-Hong Yan, Yi-Long Wu

**Affiliations:** 1 Southern Medical University, 510515 Guangzhou, Guangdong People’s Republic of China; 2 Guangdong Lung Cancer Institute, Guangdong General Hospital & Guangdong Academy of Medical Sciences, 510080 Guangzhou, Guangdong People’s Republic of China

**Keywords:** Multiple primary malignancies, Lung cancer, Clinical characteristics, Prognosis

## Abstract

**Background:**

The incidence of multiple primary malignancies (MPM) has increased sharply in recent decades. However, the clinical characteristics and prognosis of MPM patients involving lung cancer were not fully elucidated. This retrospective study was designed to explore the clinical characteristics and prognosis of MPM patients involving lung cancer in the People’s Republic of China.

**Methods:**

Of 5405 lung cancer cases diagnosed at the Guangdong Lung Cancer Institute between 2005 and 2013, we analyzed 185 patients (3.4 %) with MPM involving lung cancer.

**Results:**

Among 185 patients with MPM involving lung cancer, 10 (5.4 %)had three malignancies and 175 (94.6 %) had two malignancies. 10 patients with three malignancies were excluded from the analysis to avoid misunderstanding. Of 175 accompanying malignancies, 64 (36.6 %) were synchronous MPM patients and 111 (63.4 %) were metachronous MPM patients; 49 (28.0 %) were lung cancer first MPM patients and 126 (72.0 %) were other cancer first MPM patients. The most frequent accompanying malignancy was colon cancer (25/175), followed by rectal cancer (18/175), esophageal cancer (17/175), and thyroid cancer (13/175). Metachronous MPM patients showed significantly better overall survival (OS) than synchronous MPM, with a median OS of 72.8 (range 12.2–391.0) and 12.9 (range 0.8–86.3)months, respectively (*P <* 0.001). Cox regression analysis revealed that time of occurrence and stage were independent factors for OS.

**Conclusions:**

Colorectal cancer, esophageal cancer, and thyroid cancer were the tumors that most frequently accompanying lung cancer. Metachronous MPM patients showed significantly better OS compared with synchronous MPM patients.

## Background

Multiple primary malignancies (MPM) are defined by the presence of two or more independent primary malignancies in the same or different organs in an individual patient [1]. One of the earliest systematic studies of MPM was performed by Warren and Gates in 1932 [2]. The development of MPM is not a rare phenomenon. Based on an analysis of several studies, the incidence of MPM was estimated at 0.73–5.2 % in all tumor patients. This wide variation is related to the diverse experiences of doctors and different diagnostic tools used at different hospitals [3–5]. The incidence of MPM has increased dramatically in recent decades [6]. Lung cancer is one of the most commonly diagnosed cancers and causes the highest number of cancer-related deaths [7]. Improvements in diagnostic tools and treatment modalities, including molecularly targeted therapy, have resulted in great advances in lung cancer prognosis. Consequently, patients are surviving long enough to develop subsequent primary malignancies. Although the incidence of MPM has risen in recent decades, research on MPM involving lung cancer remains limited, especially in Chinese patients. This study retrospectively focused on the incidence, clinical features, and prognosis of MPM patients involving lung cancer at the Guangdong Lung Cancer Institute (GLCI).

## Methods

### Definition of second primary malignancy

MPM were defined according to Warren and Gate’s criteria [2]: (1) each tumor had to show definite features of malignancy; (2) each cancer had to be anatomically separate and distinct; (3) the possibility that one cancer was a recurrence or metastatic lesion of the first cancer had to be ruled out; and (4) the subsequent primary malignancies had to be present in either the same or different organs. We selected MPM patients based on the above criteria, except for cancers occurring in the same organ. MPM patients can be divided into two categories depending on the interval between tumor diagnoses. Synchronous MPM patients were defined as those occurring simultaneously or within 6 months of each other, whereas metachronous MPM patients were defined as those occurring more than 6 months apart [8]. In lung cancer first (LCF) MPM, lung cancer occurred before the secondary primary malignancy, while in other cancer first (OCF) MPM, the other primary malignancy occurred before lung cancer.

### Patients

Between January 2005 and July 2013, 185 patients at the GLCI experienced MPM involving lung cancer out of a total of 5,405 lung cancer patients. The 185 MPM patients were diagnosed comprehensively based on detailed medical history, a complete physical examination, appropriate radiographic and/or endoscopic examinations, and pathological results, which were reviewed separately by two pathologists. We arbitrarily chose 70 years as the cut-off to divide into young and old patients. Overall survival (OS) was calculated from the date of the first primary cancer diagnosis to the date of death or last follow-up of either the first or subsequent malignancy. Curative therapy was defined as treatment according to tumor classification; e.g., surgery for colorectal, gastric, lung, esophageal, cervical, breast, thyroid, and renal cancers, and radio-chemotherapy for nasopharyngeal cancer and non-Hodgkin lymphoma. Palliative treatment was defined as treatment that is non-curative but to relieve suffering, which included best supportive treatment. The patients were followed up through out-patient department visits or telephone calls. During the follow-up (median follow-up time, 41.2 months), 10 patients (5.4 %) were lost to follow-up, 76 (41.1 %) were still alive, and 99 (53.5 %) died.

### Detection of EGFR mutation status

Of 175 MPM patients with two malignancies, the EGFR mutation status of only 84 patients were reviewed. The EGFR mutation status were detected by amplification refractory mutation system(ARMS)-polymerase chain reaction or direct sequencing.

### Ethics statement

The study protocol was approved by the Institutional Review Board of Guangdong General Hospital and conformed to the ethical guidelines of the Declaration of Helsinki.

### Statistical analysis

Chi-square test, Fisher’s exact test, or a nonparametric test, where appropriate, was used for statistical comparisons. The Kaplan–Meier method was applied to univariate survival analysis. A multivariate model was built to evaluate the risk associated with prognostic factors. *P*-values less than 0.05 were considered statistically significant. All statistical analyses were performed using SPSS version 13.0 (SPSS Inc., Chicago, IL, USA).

## Results

### Patient characteristics

Of the lung cancer cases diagnosed at GLCI between January 2005 and July 2013, the incidence of MPM involving lung cancer was 3.4 % (185/5405). Of the 185 patients with MPM involving lung cancer, 175 (94.6 %) had two malignancies and 10 (5.4 %) had three malignancies. 10 patients with three malignancies were excluded from the analysis to avoid misunderstanding. There were 175 accompanying malignancies among the 175 MPM patients with lung cancer. Of the 175 accompanying malignancies, 64 (36.6 %) were synchronous MPM patients and 111 (63.4 %) were metachronous MPM patients, 49 (28.0 %) were LCF MPM patients, and 126 (72.0 %) were OCF MPM patients. Compared with metachronous MPM and OCF MPM, synchronous MPM and LCF MPM were less common. The median age at lung cancer diagnosis was 64 (range 33–88) years, and the median age at diagnosis of other cancers was 62 (range 23–89) years. In subgroup analyses, there were more young patients than old patients either in the metachronous MPM group(57.7 % vs 42.3 %, *P* = 0.05) or in the synchronous MPM group(73.4 % vs 26.6 %, *P* = 0.05). In addition, there are more cases of never smoker patients both in the metachronous MPM group(73.9 % vs 26.1 %, *P* = 0.043) and the synchronous MPM group(57.8 % vs 42.2 %, *P* = 0.043). The difference of clinical features between LCF MPM patients and OCF MPM patients did not reach significance (Table 1).

**Table 1:** Clinical characteristics of the 175 MPM patients with accompanying malignancies

Characteristics	Total *n* = 175	Synchronous group(n = 64)	Metachronous group(*n* = 111)	*P* value	LCF group(*n* = 49)	OCF group(*n* = 126)	*P* value
Age(years)				0.05			0.73
<70Y	111(63.4 %)	47(73.4 %)	64(57.7 %)		30(61.2 %)	81(64.3 %)	
> = 70Y	64(36.6 %)	17(26.6 %)	47(42.3 %)		19(38.8 %)	45(35.7 %)	
Gender				0.49			0.85
Male	125(71.4 %)	48(75.0 %)	77(69.4 %)		36(73.5 %)	89(70.6 %)	
Female	50(28.6 %)	16(25.0 %)	34(30.6 %)		13(26.5 %)	37(29.4 %)	
Smoking status				0.043			0.15
Never smoker	119(68.0 %)	37(57.8 %)	82(73.9 %)		29 (59.2 %)	90(71.4 %)	
smoker	56(32.0 %)	27(42.2 %)	29(26.1 %)		20(40.8 %)	36(28.6 %)	
Histological type				0.88			0.19
adenocarcinoma	106(60.6 %)	40(62.5 %)	66(59.5 %)		27(55.1 %)	79(62.7 %)	
Squamous cell carcinoma	45(25.7 %)	16(25.0 %)	29(26.1 %)		18(36.7 %)	27(21.4 %)	
SCLC	17(9.7 %)	5(7.8 %)	12(10.8 %)		3(6.1 %)	14(11.1 %)	
Other NSCLC	7(4.0 %)	3(4.7 %)	4(3.6 %)		1(2.0 %)	6(4.8 %)	
Stage for lung cancer				0.34			0.076
I	42(24.0 %)	20(31.3 %)	22(19.8 %)		10(20.4 %)	32(25.4 %)	
II	29(16.6 %)	10(15.6 %)	19(17.1 %)		8(16.3 %)	21(16.7 %)	
III	38(21.7 %)	14(21.9 %)	24(21.6 %)		17(34.7 %)	21(16.7 %)	
IV	66(37.7 %)	20(31.3 %)	46(41.4 %)		14(28.6 %)	52(41.3 %)	
Tumor interval Median(range)	19.3(0.00-391.0)	0.52(0.00-5.1)	49.0(6.03-391.0)	<0.001	2.07(0.00-152.2)	36.0(0.00-391.0)	<0.001

### Malignancies accompanying lung cancer

Of the 175 accompanying malignancies, the most frequent accompanying malignancy was colon cancer (25/175), followed by rectal cancer (18/175), esophageal cancer (17/175) and thyroid cancer (13/175). The patients comprised 125 men and 50 women, giving a sex ratio of 2.5:1. The different types of accompanying malignancies are presented in Table 2. There were 67 accompanying malignancies that received curative treatment, 108received palliative treatment, The curative treatment rate of accompanying malignancies was only 38.3 %(67/175). There were 122 accompanying malignancies that underwent surgery, and the percentage of accompanying malignancies addressed with operation versus non-surgery were 64.6 %(113/175) and 35.4 %(62/175), respectively. For accompanying malignancies of the digestive system, there were significantly more LCF MPM patients than OCF MPM patients (32/49,65.3 % vs. 48/126,38.1 %, *P* = 0.001) and significantly more synchronous cases than metachronous cases (37/64,57.8 % vs. 43/111,38.7 %, *P* = 0.018). For accompanying malignancies in the urogenital system, there were more metachronous cases than synchronous cases (26/111,23.4 % vs. 7/64,10.9 %, *P* = 0.046) and more OCF MPM patients than LCF MPM patients (30/126,23.8 % vs. 3/49,6.1 %, *P* = 0.009; Table 2).

**Table 2:** Malignancies accompanying lung cancer

Sites	Total(*n* = 175)	Synchronous group(*n* = 64)	Metachronous group(*n* = 111)	*P-value*	LCF group(*n* = 49)	OCF group(*n* = 126)	*P-*value
Digestive system	80(45.7 %)	37(57.8 %)	43(38.7 %)	0.018	32(65.3 %)	48(38.1 %)	0.001
Colon	25	9	16		5	20	
Rectum	18	5	13		7	11	
Esophagus	17	8	9		5	12	
Liver	9	6	3		6	3	
Stomach	9	9	0		9	0	
Duodenal	1	0	1		0	1	
Gallbladder	1	0	1		0	1	
Urogenital system	33(18.9 %)	7(10.9 %)	26(23.4 %)	0.046	3(6.1 %)	30(23.8 %)	0.009
Urinary bladder	10	2	8		1	9	
Prostate	5	0	5		1	4	
Uterine cervix	7	1	6		0	7	
Kidney	4	1	3		1	3	
Ovary	4	2	2		0	4	
Endometrium	3	1	2		0	3	
Head&Neck	34(19.4 %)	12(18.7 %)	22(19.8 %)	1.00	9(18.4 %)	25(19.8 %)	0.84
Thyroid gland	13	6	7		6	7	
Larynx	8	2	6		2	6	
Nasopharynx	9	2	7		0	9	
Parotid gland	1	1	0		0	1	
Tongue	2	0	2		0	2	
Soft palate	1	1	0		1	0	
Lymphatic&hemat-opoietic system	11(6.3 %)	4(6.3 %)	7(6.3 %)	1.00	2(4.1 %)	9(7.2 %)	0.73
Lymphoma	8	3	5		1	7	
Leukemia	3	1	2		1	2	
Others	17(9.7 %)	4(6.3 %)	13(11.7 %)	0.30	3(6.1 %)	14(11.1 %)	0.41
Breast	10	3	7		1	9	
Skin	5	1	4		1	4	
Nasal olfactory cell	1	0	1		0	1	
Osteosarcoma	1	0	1		1	0	

### Univariate and multivariate analyses of prognosis

**Fig. 1:**
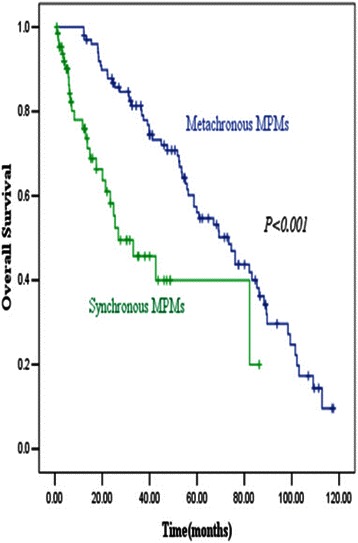
Metachronous MPM patients demonstrated significant better OS than synchronous MPM patients, with median OS rates of 72.8 (range 12.2–391.0) and 12.9 (range 0.8–86.3) months, respectively (*P* < 0.001), which suggests that metachronous MPM patients have a better prognosis than synchronous MPM patients

The 1- and 5-year OS of patients with 175 MPM involving lung cancer were 92 % and 59 %, respectively. The 5-year OS rates for patients with metachronous and synchronous MPM were 68 % and 38 % respectively (*P* < 0.001). Metachronous MPM patients demonstrated significantly better OS than synchronous MPM patients (Fig. 1), with a median OS of 72.8 (range 12.2–391.0) and 12.9 (range 0.8–86.3) months, respectively (*P* < 0.001). The median OS rates were 39.8 (range 0.8–391.0) months and 43.1 (range 3.9–341.9) months in male and female MPM patients, respectively; the difference was not significant between sexes (*P* = 0.15). The effects of clinical factors on prognosis were also evaluated. Higher stage, time of occurrence (synchronous MPM), and order of occurrence (LCF MPM) were significantly associated with poorer OS. Moreover, Cox regression modeling revealed that the time of occurrence and stage were independent factors for OS (Table 3). We split the MPM patients based on the order of occurrence to compare OS between the metachronous and synchronous patients. In LCF MPM patients, OS between metachronous MPM patients and synchronous MPM patients did not reach statistical significance (*P* = 0.31), with a median OS of 47.5(range 12.2–243.7) months and 13.0 (range 0.8–48.7) months, respectively. In OCF MPM patients, metachronous MPM patients showed a statistically significant improvement in OS than synchronous MPM patients (*P* < 0.001), with a median OS of 76.1 (range 12.2–391.0) months and 12.7 (range 0.9–86.3) months, respectively.

**Table 3:** Univariate and multivariate survival analysis of prognostic factors in MPM patients

Characteristics	OS rate (%)		Univariate	Multivariate	
	1Y	5Y	*P* value	HR(95 % CI)	*P* value
Age(years)			0.95	1.16(0.61-2.21)	0.65
<70Y	94 %	68 %			
> = 70Y	91 %	67 %			
Gender			0.15	1.05(0.54-2.03)	0.89
Male	90 %	66 %			
Female	98 %	70 %			
Smoking status			0.28	0.74(0.38-1.45)	0.38
Never smoker	95 %	70 %			
Smoker	88 %	63 %			
Stage for lung cancer			<0.001	1.66(1.38-2.03)	<0.001
I	93 %	91 %			
II	100 %	79 %			
III	95 %	55 %			
IV	88 %	55 %			
Treatment for other cancers			0.14	1.21(0.75-1.95)	0.44
Curative	94 %	75 %			
Palliative	92 %	63 %			
Histological type			0.16	1.14(0.92-1.43)	0.24
Adenocarcinoma	93 %	69 %			
Squamous cell carcinoma	93 %	67 %			
SCLC	88 %	53 %			
Other NSCLC	100 %	86 %			
Time of occurrence			<0.001	0.19(0.10-0.36)	<0.001
Synchronous	80 %	61 %			
metachronous	100 %	71 %			
Order of occurrence			0.047	0.85(0.49-1.47)	0.56
LCF	88 %	63 %			
OCF	94 %	69 %			

*OS* Overall Survival; *HR* Hazard Ratio; *CI* Confidence Interval

### EGFR mutation detection

Of 175 MPM patients, there were only 84 patients who had EGFR mutation status, 25 cases were detected by the methods of direct sequencing while 59 cases were detected by the methods of ARMS, There are only 84 patients subjected to EGFR mutation analysis because EGFR mutation analysis was not becoming the routine clinical practice until 2009, what’s more, the quality and quantity of a portion of lung cancer specimens was unqualified or not enough for the EGFR mutation analysis and these patients denied re-biopsy or the performance status(PS) was not suitable for re-biopsy. Of the 84 patients, there were only 24 patients with EGFR mutations for an EGFR mutation rate of only 28.6 %.

## Discussion

MPM incidence has increased significantly owing to advances in diagnostic methods and new therapies, such as targeted therapies, which allow more patients to survive long enough to develop subsequent primary tumors [9, 10], screening procedures are useful for the early detection of possible MPM, especially for the patients who are diagnosed in advanced stages [11]. Although there are numerous reports addressing the clinical features of MPM, only a few studies in Taiwan and Japan have investigated MPM involving lung cancer [12, 13]. Unfortunately, these reports have only marginally improved our understanding of the clinical features of patients with this disease. Therefore, this retrospective study was designed to evaluate the clinical characteristics and outcomes of MPM patients involving lung cancer in Chinese patients. The incidence of MPM patients involving lung cancer in our study was 3.4 % (185/5405), similar to the results of a previous study from Turkey, which found an incidence of 3.9 % (40/1038) [14]. The most frequent accompanying malignancies were colon, rectal, and esophageal cancers, which suggests that lung cancer may be closely associated with digestive system neoplasms. The next most frequent accompanying malignancies were thyroid, liver, breast, urinary bladder, and laryngeal cancers, a distribution similar to cancers in the general Chinese population. Rare accompanying malignancies, such as cancers of duodenal, gallbladder, tongue, soft palate, nasal olfactory cell, and osteosarcoma, were also identified. In addition, the observed male predominance, with a sex ratio of 2.5:1 for MPM patients, is consistent with a previous report of male/female ratios ranging from 0.9:1 to 3.5:1 [15]. Moreover, there were 119 non-smokers (68 %) and 56 smokers (32 %), which suggests that unknown factors other than tobacco play an important role in the increased incidence of MPM. It is well established that a high incidence of epidermal growth factor receptor (EGFR) mutations is associated with clinical features, such as adenocarcinoma histology, Asian ethnicity, female sex, and never-smoker status [16–18], and MPM patients with nearly 60 % adenocarcinoma histology and nearly 70 % never-smoker status indicate the high possibility of EGFR mutations, which might explain the increased incidence of MPM patients involving lung cancer. Based on these results, we reviewed the EGFR mutation status of lung cancer specimens, however, we did not have complete EGFR mutation data because our sample set included early records from when EGFR mutation detection was not routine clinical practice. In fact, there were only 84 patients who had EGFR mutation status detected by amplification refractory mutation system-polymerase chain reaction and direct sequencing, and there were only 24 patients with EGFR mutations for an EGFR mutation rate of only 28.6 %. This is similar to the common Asian population, which suggests that EGFR mutations are not responsible for the increased incidence of MPM patients involving lung cancer.

In the univariate analysis, we demonstrated no OS difference between male and female patients, which is different from one reported study showing better OS in female patients [10]. The discrepancy may be the result of the different types of tumors and heterogeneity of the study populations. Our study suggests that time of occurrence and stage are independent factors for OS in MPM patients. Metachronous MPM patients had a better prognosis than synchronous MPM patients. This is consistent with previous studies of MPM involving lung cancer [12] and MPM involving other cancers, such as gastric cancer and hepatocellular carcinoma [19–21], and it is because of detection of the second primary cancer at a curable stage with periodic medical check-ups for patients with a history of cancer. However, there were 70 patients (63 %) in the metachronous group with stage III or IV lung cancer and only 34 patients (53.2 %) in the synchronous group. We believe that the following reasons can explain this contradiction. First, time of occurrence other than stage is the most important determining factor of MPM patient prognosis, which was shown in the multivariate analysis. Second, there were 18 LCF MPM patients and 93 OCF MPM patients in the metachronous group. More OCF MPM patients were observed in the metachronous group, which suggests a better prognosis.

There are several studies focusing on the difference between synchronous and metachronous groups, such as MPM involving lung cancer and MPM involving gastric cancer and hepatocellular carcinoma, however, to the best of our knowledge, only one study has focused on the difference between LCF and OCF groups [12], which did not show a significant difference either calculated time from the diagnosis of the first cancer or from the second cancer. In this study, the prognosis did not show a significant difference between LCF and OCF MPM patients on multivariate analysis, which is consistent with the previous study. Based on the order of occurrence, we split MPM patients to compare the OS between the metachronous and synchronous patients either in LCF MPM or in OCF MPM patients. Our results showed that metachronous MPM patients have better OS than synchronous MPM patients in the OCF MPM group, but it did not show a significant difference in the LCF MPM group, which suggests that lung cancer is more aggressive than other malignancies. In addition, Zeng et al. reported no difference in OS between patients with MPM involving hepatocellular carcinoma (HCC) and patients with HCC alone [22], indicating that extrahepatic primary malignancies had no effect on the survival of HCC patients. For these reasons, MPM patients do not always have a poor prognosis, especially patients with metachronous MPM.

Although the underlying mechanisms of MPM have not been fully elucidated, inherited predisposition is thought to be an important factor [23]. Such predispositions include Lynch syndrome, an autosomal dominant-inherited disorder of colorectal cancer, and susceptibility to other tumors caused by germline mutations in DNA mismatch repair genes [24]. The immune system of patients was suggested to be another important factor [25], and intensive exposure to carcinogens including chemotherapy and/or radiotherapy used in the treatment of tumors [26, 27] and field cancerization in organs exposed to carcinogens, leading to the proliferation of numerous primary tumors, have also been suggested to be responsible for MPM [28, 29].

Recently, “ALKoma” was proposed as a cancer subtype with a shared target as an essential growth driver [30]. More and more investigators suggest that cancers of different organs of origin, but with the same molecular targets, should be managed together because the common molecular targets observed in diverse tumors determine clinical practice better than organ-based classification [31]. Consequently, further genetic and molecular studies using next-generation sequencing explored whether MPM involving lung cancer share common molecular targets, such as EGFR, Kirsten rat sarcoma viral oncogene homolog (KRAS) and anaplastic lymphoma kinase (ALK) to improve therapeutic outcome. Further genetic and molecular investigations will focus on understanding the pathogenesis of MPM involving lung cancer and improving therapeutic outcomes.

There are two new features in the present study that differ from previous reports. First, this study had the largest number of MPM cases involving lung cancer in mainland People’s Republic of China. Second, it is the first study to explore the impact of both time of tumor occurrence (synchronous MPM vs. metachronous MPM) and order of tumor occurrence (LCF MPM vs OCF MPM) on the survival of MPM patients. Our results show that metachronous MPM patients have a better prognosis compared with synchronous MPM patients, 6 months intervals may partially explained why metachronous MPM patients have a better prognosis than synchronous MPM patients.

Although our study revealed some unique results, there are some potential shortcomings and limitations. First, the study was retrospective and was conducted at a single institution. Second, the study did not explore the impact of extrapulmonary primary malignancies on lung cancer survival. Third, some information was deficient because of the long follow-up duration. Nonetheless, the consistency with reported studies confirms the validity of our conclusions.

## Conclusions

Colorectal cancer, esophageal cancer, and thyroid cancer were the tumors most frequently accompanying lung cancer. Time of occurrence and stage were independent factors for OS, which suggests that MPM patients presenting with metachronous cancers and early stage have a better prognosis.

## Abbreviations

MPM: Multiple primary malignancies
LCF: Lung cancer first
OCF: Other cancer first
OS: Overall survival
EGFR: Epidermal growth factor receptor
KRAS: Kirsten rat sarcoma viral oncogene homolog
ALK: Anaplastic lymphoma kinase
HCC: Hepatocellular carcinoma
BST: Best support treatment
